# Editorial: Intraoperative Visualization Techniques and Advanced Imaging in Brain Tumors

**DOI:** 10.3390/cancers18020298

**Published:** 2026-01-19

**Authors:** Andrea Bianconi, Pietro Fiaschi, Diego Garbossa

**Affiliations:** 1 Division of Neurosurgery, Ospedale Policlinico San Martino, IRCCS for Oncology and Neurosciences, Largo Rosanna Benzi 10, 16132 Genoa, Italy; 2 Department of Neuroscience, Rehabilitation, Ophthalmology, Genetics and Maternal and Child Health, University of Genoa, Largo Rosanna Benzi 10, 16132 Genoa, Italy; 3 Neurosurgery Unit, Department of Neuroscience, University of Turin, Via Cherasco 15, 10126 Turin, Italy

On behalf of the Editors, we wish to express our sincere gratitude to all authors and reviewers who contributed to the success of this Special Issue, titled “Intraoperative Visualization Techniques and Advanced Imaging in Brain Tumors”.

The primary objective of surgical neuro-oncology, particularly concerning primary brain tumors, has long been defined by the pursuit of Maximal Safe Resection: the most radically oncological removal of tumor tissue possible, while simultaneously preserving the patient’s neurological integrity [1,2,3]. This goal, now the standard of care, is currently underpinned by a rapidly evolving ecosystem of advanced technologies [4,5,6].

## 1. The Contributions of the Special Issue

This Special Issue aimed to provide a focused update on the technologies currently available, offering the neurosurgical, neuroradiological, and neuro-oncological communities a rigorous and synthetic overview. The seven published contributions, consisting of five Original Research articles and two Reviews, reflect the dual and complementary nature of the challenge: pre-operative visualization and intra-operative guidance. The articles collected herein span the spectrum from advanced in vivo imaging biomarkers to clinical outcome analyses and novel intraoperative integration strategies.

Focusing on diagnostic and prognostic biomarkers, the work by Onishi et al. utilized Chemical Exchange Saturation Transfer (CEST) imaging and Diffusion-Weighted Imaging (DWI) on a rat glioma model. Their findings demonstrated that X-ray irradiation decreased certain MTR values (e.g., those corresponding to creatine) and inhibited the increase in ADC standard deviation, suggesting potential imaging biomarkers for assessing treatment response.

The critical importance of advanced pre-operative planning and cognitive network analysis was highlighted by the two comprehensive reviews. Vintimilla Rivadeneira et al. synthesized evidence on multimodal strategies for function-guided intervention, emphasizing the integration of fMRI, DTI, tractography, and Direct Electrical Stimulation (DES) to preserve cognitive and neurological function during resection. Expanding this functional perspective, the narrative review by Martínez Lozada et al. explored the impact of intracranial tumors on large-scale neural networks via connectomic analysis (using rs-fMRI and diffusion MRI), underscoring that functional heterogeneity demands a “network-aware” approach to surgical planning.

In the realm of intraoperative guidance, fluorophore application was addressed in two original papers. Pesaresi et al. reported on a case series of 100 glioblastoma resections, demonstrating the efficacy of a combined approach using 5-ALA and sodium fluorescein (SF). This dual-fluorescence technique achieved Gross Total Resection (GTR) in a high percentage of cases by leveraging the distinct advantages of each agent. Furthermore, Matsuda et al. investigated the utility of 5-ALA fluorescence beyond gliomas, conducting a prospective clinical trial that confirmed its role in improving the extent of resection for invasive intracranial meningiomas.

Addressing other key intraoperative challenges, the paper published by Bopp et al. focused on enhancing surgical guidance in the sitting position, a scenario often complicated by brain shift. Their research demonstrated that image-based co-registration of pre-operative MRI and Intraoperative Ultrasound (iUS) significantly improved navigation accuracy, thereby enabling the reliable use of navigation and Augmented Reality (AR) in posterior fossa surgery. Finally, Pauletto et al. contributed an important clinical outcome study, determining that Intraoperative Seizures (IOS) do not impact long-term seizure control or clinical outcomes, while drawing attention to the higher impact of early Post-operative Seizures (POS) in the first year following glioma surgery.

## 2. Advanced Pre-Operative Planning

Several studies within this collection emphasized the critical role of advanced diagnostic imaging in the surgical planning phase. The integration of functional Magnetic Resonance Imaging (MRI) techniques—both resting-state and task-based—and advanced models of tractography (Diffusion Tensor Imaging, DTI) and fiber tracking now allows for patient-specific structural and functional mapping of the brain [7,8,9,10]. Such tools are fundamental in defining the safety margins of the resection, particularly for lesions located near eloquent areas. Furthermore, the growing role of Artificial Intelligence (AI) emerges as a significant trend, notably for automated tumor segmentation and optimizing surgical trajectories [11,12,13].

## 3. Intraoperative Guidance Technologies

The second main theme addressed concerns real-time visualization techniques, which are essential for overcoming challenges related to brain shift and for distinguishing neoplastic tissue from healthy parenchyma. The contributions explored the use of fluorophores—such as 5-ALA (Gliolan) and sodium fluorescein, [14,15,16] in combination or not with intraoperative confocal microscopy—which enhance the contrast between the tumor and the surrounding parenchyma [17,18,19,20]. Concurrently, the relevance of integrated imaging methodologies is highlighted, such as Intraoperative Ultrasound and the use of Intraoperative MRI or CT, which allow for updating surgical navigation during the procedure.

## 4. Future Perspectives

The variety of techniques and technologies presented in the Special Issue reflects the current challenge faced by neurosurgeons, neuroradiologists, and neuro-oncologists: selecting the optimal toolset within a vast, dynamic, and rapidly evolving landscape. The availability, required surgical skills, and comparative efficacy of these methodologies vary significantly across different centers. This Special Issue serves as an additional piece of information intended to facilitate orientation within this complex scenario. The effectiveness of Maximal Safe Resection does not depend on the adoption of a single technology, but on the surgical team’s ability to selectively and wisely integrate the different tools available, creating a personalized, multimodal approach for each patient.

In conclusion, the evolution of visualization and advanced imaging techniques continues to redefine the boundaries of safe resectability. The future of surgical neuro-oncology lies in the further integration of AI, advanced imaging, and intraoperative guidance to translate increased knowledge into a tangible improvement in patients’ neurological and oncological outcomes.
